# Helleborus Albus Used for Producing Artificial Cardiac Disease

**Published:** 1829-07-01

**Authors:** 


HELLEBORUS ALBUS USED FOR PRODUCING ARTIFICIAL CARDIAC DISEASES.

Dr. Quarrier has given a short notice to the above effect, in the first number of the Provincial Medical Gazette. A man of the name of Chapman, belonging to the marine artillery, had found out the secret virtues of the white hellebore, and turned it to the advantage (or rather disadvantage) of himself and others, to whom he sold his powders at a high price. By taking the hellebore in various doses, the effects were, "either a sudden and severe illness for the immediate occasion, or to gradually undermine the tone of the stomach and digestive organs, producing every appearance of dyspepsia, attended by great nervous irritability, and violent and continued palpitation of the heart." This Chapman had deserted, and was taken in a remote part of the country, where he completely succeeded in deceiving the staff-surgeon of the district militia, who examined him, and reported his "total incapacity for service, in consequence of *organic disease of the heart.*"

The foregoing facts are not uninteresting, because they shew how much the function, or at least the action of the heart is influenced by the state of the stomach. Dr. Quarrier states another fact which is worthy of attention. "This practice (of taking hellebore) was productive of some alarming consequences for a considerable period :—some were permanently injured, having actually produced the disease which they intended to counterfeit." Thus disorder of function led to disease of structure in many instances. It is curious, however, that white hellebore should so peculiarly affect the heart when taken into the stomach. We can hardly attribute this solely to sympathy of the one organ with the other. It would seem that there is a special or specific tendency in hellebore to affect the heart, as squill affects the kidneys, or cubebs the urethra. There is no accounting for, nor yet denying these specific tendencies in drugs. On the knowledge of these specialities most of our success in practice depends.

---

XXII.

